# Stop signals decrease choices for palatable foods through decreased food evaluation

**DOI:** 10.3389/fpsyg.2013.00875

**Published:** 2013-11-26

**Authors:** Harm Veling, Henk Aarts, Wolfgang Stroebe

**Affiliations:** ^1^Department of Psychology, Radboud University NijmegenNijmegen, Netherlands; ^2^Department of Psychology, Utrecht UniversityUtrecht, Netherlands; ^3^Department of Psychology, University of GroningenGroningen, Netherlands

**Keywords:** motor inhibition, appetite, food evaluation, stop signals, go/no-go task

## Abstract

The present study explores whether presenting specific palatable foods in close temporal proximity of stop signals in a go/no-go task decreases subsequent evaluations of such foods among participants with a relatively high appetite. Furthermore, we tested whether any decreased evaluations could mediate subsequent food choice. Participants first received a go/no-go task in which palatable foods were consistently linked to go cues or no-go cues within participants. Next, evaluation of the palatable foods was measured as well as food choice. Replicating previous work, results show that among participants with a relatively high appetite palatable foods associated with no-go cues are less often chosen as snacks compared to when these foods are associated with go cues, whereas this manipulation did not affect participants with a relatively low appetite. Moreover, this effect was completely mediated by decreased evaluation of the palatable foods that had been associated with the no-go cues, whereas evaluation of the foods associated with go cues did not mediate this effect. Results further showed that the devaluation effect of foods associated with no-go cues was independent of the amount of pairings (4 vs. 12 vs. 24) with the no-go cues. The current findings suggest that decreased food evaluation is a mechanism that explains effects of stop signals on food choice.

## INTRODUCTION

Influencing people’s choices for specific foods is an important topic of scientific inquiry, because the profound influence of food choice on maintaining a healthy body weight. Based on insights that eating behavior in general, and food choice in particular, occurs often rather mindlessly (Wansink, 2004), recent work focuses on ways to influence food choice without relying on people’s deliberate decision making, but by influencing impulsive or automatic determinants of food choice (Hofmann et al., 2008). For instance, recent work suggests that linking images of aversive health consequences of eating too many calories to specific foods can decrease subsequent choices for such foods (Hollands et al., 2011). The idea behind this approach is that by directly modifying psychological responses toward specific foods, food choice can be affected even if people do not think all that much about their choices (Strack and Deutsch, 2004; Sheeran et al., 2013).

One way of changing people’s impulsive or automatic behavior toward foods or drinks is by presenting pictures of such stimuli during a short training^1^ procedure in which participants responses toward the stimuli are manipulated (e.g., Wiers et al., 2011). Recent research suggests that one effective way of modifying immediate responses toward specific foods or drinks is by linking these stimuli to behavioral stop signals in a go/no-go task (Veling and Aarts, 2011a). Specifically, consistently presenting palatable foods with no-go cues in a go/no-go task has been shown to reduce subsequent consumption of these foods especially among people who find these foods hard to resist (Houben, 2011; Houben and Jansen, 2011; Veling et al., 2011). Apart from affecting behavior toward food, stop signals have also been shown to reduce consumption of alcoholic beverages among heavy drinkers (Houben et al., 2011; Jones and Field, 2013). Moreover, research has shown that stop signals not only reduce quantity of consumption, but can also decrease choices for specific palatable foods (Veling et al., 2013). In the present research we aimed to gain new insight into this topic by examining the psychological mechanism that may underlie this decreased choice effect.

Although studies examining the mechanism by which stop signals exert an effect on consumption behavior are rare (for an exception see Houben et al., 2012), several studies have found moderators that specify conditions under which the stop signals exert an effect on behavior. Specifically, the stop signals during the go/no-go task appear effective in reducing subsequent behavioral responses toward foods and drinks, and even sexually appealing opposite sex others, particularly when these stimuli are rewarding or have high incentive value (Veling and Aarts, 2009; Houben and Jansen, 2011; Ferrey et al., 2012). For instance, in the context of food choice it has been shown that stop signals reduced choices for specific palatable foods when participants had a relatively high appetite (i.e., as defined as a natural desire to satisfy a bodily need, especially for food; e.g., before lunch), but not when they had a relatively low appetite (e.g., after lunch; Veling et al., 2013). People are more sensitive for the impulse evoking qualities of food when the incentive value of food as a result of appetite is high, as evidenced by increased impulsive reactions toward the foods (Seibt et al., 2007; see also Tuorila et al., 2001; Nederkoorn et al., 2009). Stop signals that inhibit such impulsive reactions can therefore be expected to be effective under this condition. However, the psychological mechanism through which reduced choices for specific foods occur under the condition of high appetite was not examined in this previous work.

There are at least two possible mechanisms that may explain how stop signals may reduce choices for specific palatable foods among participants with a relatively high appetite. First, it could be that the manipulation creates a link between a stimulus and the goal of stopping behavior toward that stimulus (Verbruggen and Logan, 2008). Once such a link is established, the stop goal may be activated in a bottom-up fashion when the food is subsequently encountered in a choice situation (i.e., upon perception of the food), and bias choices toward other available options. A second mechanism that may explain why palatable foods that have been associated with no-go cues are subsequently less chosen is that these foods are devalued, i.e., their perceived attractiveness or incentive value is decreased (Fenske and Raymond, 2006; Veling and Aarts, 2009; Ferrey et al., 2012). According to Behavior Stimulus Interaction (BSI) theory (Veling et al., 2008) this devaluation occurs to release the approach impulse toward the food, and hence prevent possible continuous oscillation between approach triggered by the food and inhibition triggered by the stop signal. Although evaluations of palatable foods after the go/no-go task have not yet been examined, support for the hypothesis that stop signals may reduce evaluations of these foods stems from other work that has found that attractive stimuli are devalued after repeated association with no-go cues. Based on this work we currently focused on this second potential mechanism.

Specifically, research using general positive affective pictures has shown that evaluations of these pictures are decreased after they have been presented with no-go cues compared to both pictures associated with go cues and new pictures (Veling et al., 2008; Frischen et al., 2012). This and other work suggests that go responses do not affect subsequent evaluations to positive stimuli whereas no-go cues reduce such evaluations (for an overview see Fenske and Raymond, 2006). This affective devaluation effect has proven quite robust as it has been found for different types of attractive stimuli such as alcoholic beverages for heavy drinkers (Houben et al., 2012), and erotic images (Ferrey et al., 2012). In the present work we were interested to (a) test whether no-go cues can also devaluate evaluations of palatable foods, (b) whether this effect is dependent on people’s appetite and the amount of pairings with no-go cues, and (c) whether any devaluation of palatable foods would mediate subsequent decreases in choices for such food.

### OVERVIEW OF THE CURRENT STUDY

To address these questions participants with low vs. high appetite first received a go/no-go task in which three pictures of palatable foods were consistently presented together with go cues (go foods), and three pictures of palatable foods with no-go cues (no-go foods). Unlike previous work that examined the effectiveness of this manipulation on food choice (Veling et al., 2013), we currently employed a within-subject manipulation of the go/no-go task. This design was employed to be able to pick up any subtle differences in evaluations between the go and no-go foods. Accordingly, after the go/no-go task participants were asked to rate the attractiveness of these foods, and they were asked to choose three snacks that they would like to consume. We expected decreased evaluations of no-go compared to go foods, and that this devaluation effect would mediate choice behavior. With regard to the question of the amount of pairings that may be necessary to obtain a devaluation effect no systematic tests have been reported to date. For exploratory reasons, we used three levels of amounts of pairing with go and no-go cues (4 vs. 12 vs. 24) within participants.

## MATERIALS AND METHODS

### ETHICS STATEMENT

The study was conducted, and written informed consent of each participant was obtained in compliance with the principles contained in the Declaration of Helsinki.

### PARTICIPANTS AND DESIGN

Fifty participants (34 women; mean age 22) participated in this study for course credit or a small payment. Participants were assigned to the high appetite condition when they participated before lunch and to the low appetite condition when they participated after lunch (e.g., Seibt et al., 2007; Veltkamp et al., 2008). We used this operationalization of appetite because it allowed us to examine whether stop signals are effective under everyday circumstances where people’s appetite is relatively high (i.e., before lunch; e.g., de Graaf et al., 1993; Blundell et al., 1996). Moreover, this operationalization of appetite has been used in previous work (e.g., Seibt et al., 2007; Veling et al., 2013). The main research design is a 2 (food status; go vs. no-go) by 3 (amount of pairings: 4 vs. 12 vs. 24) by appetite (low vs. high) mixed design with food status and amount of pairings as within-subject factors and appetite as between-subject factor.

### STIMULI

The seven pictures of palatable foods used in this study are taken from a previous study that confirmed that these foods are perceived as palatable (i.e., chocolate bar, potato chips, chocolate muffin, M&M’s, almond paste cookies, chocolate chip cookies; for the pictures and previous ratings of these foods see Veling et al., 2013). Six different food stimuli were allocated to a 2 (food type: go vs. no-go) × 3 (amount of pairings: 4 vs. 12 vs. 24) within subjects design during the go/no-go task (one specific food stimulus in each cell). The remaining picture was only presented after this manipulation. This latter picture (called new picture) was included to provide a neutral baseline for the picture evaluations. The function of each picture (go vs. no-go vs. new; 4 vs. 12 vs. 24 presentations in the go/no-go task) was counterbalanced across participants.

### GO/NO-GO TASK

The go/no-go task was presented as a study on how fast people can direct their attention. This task in fact contained the manipulations of food status and amount of pairings. Participants learned that pictures would be presented on a computer screen together with the letter A or L. These letters were presented in one of the four quadrants of the pictures, and these picture-letter combinations were presented for 1500 ms. Immediately after this presentation a blue question mark would appear, and the task of the participant was to press when the letter A (or L counterbalanced across participants) had been presented and to refrain from responding when the letter L (A) had been presented. Some pictures were always paired with an instruction to respond (go cues), and other pictures were always paired with the instruction to withhold responding (no-go cues). The question mark remained on screen for 1000 ms or until a response was detected. After a correct response a green circle was presented, and after an incorrect response a red cross was presented for 500 ms. The intertrial interval was 500 ms.

Participants were presented with six different food pictures during the go/no-go task that were consistently presented according to one of the cells of the 2 (food status: go vs. no-go) by 3 (amount of pairings: 4 vs. 12 vs. 24) design. The go/no-go task thus consisted of a total of 80 trials. Trials were presented in a random order.

### MEASURES

After the go/no-go task participants learned that we needed evaluations of pictures for future research. They were asked to rate pictures on a scale ranging from 1 (not at all attractive) to 7 (very attractive). The seven palatable food pictures were then presented in random order. After that, participants read that they could select three snacks for consumption. They were presented with pictures of the seven palatable foods selected for this study, and they could select three of them by clicking on them with the mouse. We did not inform participants that the choice was hypothetical, but we also did not provide them with information about the consequences of their choice. Next, participants rated the pictures on palatability on a scale that ranged from 1 (not at all tasty) to 9 (very tasty), and on frequency of consumption (“How often do you consume X”) on a scale ranging from 1 (not at all often) to 9 (very often). Finally, we collected information with regard to participants’ appetite (whether they participated before or after lunch). We also measured time since last food consumption in minutes, and self-reported appetite on a nine-point scale ranging from not at all to very high. We collected these data after rather than before the manipulation and dependent variables in order to not sensitize participants to the purpose of the study before the manipulation. Next, we measured participants’ intentions to eat healthily, administered the chronic dieting scale (Herman and Polivy, 1980), we measured perceived dieting success (Fishbach et al., 2003) asked for participants’ weight and height, and collected demographical data.

### PROCEDURE

Participants were greeted by an experimenter who told participants that all instructions for the study would appear on a computer screen. Participants performed the study individually in cubicles. They started with the go/no-go task, which was followed by the measures. Afterward participants were debriefed and they received a small payment.

## RESULTS

Analyses were performed using SPSS 20.

### PARTICIPANT CHARACTERISTICS AND MANIPULATION CHECKS

Participants in the low vs. high appetite conditions did not differ with regard to % of women, age, chronic dieting scores (overall *M* = 6.46; SD = 3.44), perceived dieting success (overall *M* = 4.55; SD = 1.47; Fishbach et al., 2003), body mass index (BMI; overall *M* = 22.48; SD = 3.74; one participant did not report her weight), intentions to eat healthily (overall *M* = 7.00; SD = 1.36), and frequency of consuming the foods used in the present study (overall *M* = 4.47; SD = 1.07), *F*s < 1.6, *p*s > 0.22. Moreover, accuracy in the go/no-go task was high (*M* = 0.98; SD = 0.05), indicating that participants performed the task well, and accuracy did not differ between appetite conditions, *F*(1,48) = 2.11, *p* = 0.15, η^2^ = 0.04. Mean reaction times on correct go trials (*M* = 156; SD = 30.02) did also not differ between appetite conditions, *F* < 1.

Because we assessed people’s appetite only after the manipulation and collecting the dependent variables, we did not have control over the number of participants in each appetite condition. In the current study in turned out that 19 participants participated before lunch and 31 participated after lunch. Manipulation check revealed that self-reported appetite was higher in the high appetite condition (*M* = 5.84; SD = 2.03) compared to the low appetite condition (*M* = 4.06; SD = 2.03), *F*(1,48) = 9.01, *p* < 0.01, η^2^ = 0.16. Moreover, time since last food consumption (in minutes) was also higher in the high appetite condition (*M* = 210; SD = 199) compared to the low appetite condition, (*M* = 66; SD = 52), *F*(1,48) = 14.68, *p* < 0.01, η^2^ = 0.23.

Repeating the analyses reported below by using a median split on time since last food consumption (creating a high appetite group of *N* = 25 and a low appetite group of *N* = 25) led to similar significant results as when the appetite variable based on people’s lunch was used, except that the effect of food status (go vs. no-go) by appetite interaction on food choice dropped to marginally significant (*p* = 0.051). However, repeating the analyses with self-reported appetite as measured on the nine-point scale did not lead to similar significant results as when the appetite variable based on people’s lunch was used.

### FOOD EVALUATION

In designing the study we intended to use the attractiveness ratings before the choice measure as a measure of evaluation, and use the tastiness ratings after the choice to confirm that our foods were indeed perceived as tasty by our participant group. As it turned out, the two evaluation measures reacted similar to our manipulations. Furthermore, the overall correlation between the ratings was high (*r* = 0.92), and the significance of the analyses reported below is not changed when we use only the attractiveness ratings, or only the tastiness ratings. Therefore, we decided to use as a measure of food evaluation the mean of the attractiveness and tastiness rating of each picture.

To test whether the no-go foods were evaluated less positively than the go foods independent of the amount of pairings, we conducted a 2 (food status; go vs. no-go) by 3 (amount of pairings: 4 vs. 12 vs. 24) by appetite (low vs. high) mixed measures general linear model (GLM) with food status and amount of pairings as within-subject factors and appetite as between-subject factor on food evaluation. This analysis revealed a main effect of food status, *F*(1,48) = 4.89, *p* = 0.03, η**_p_^2^ = 0.09, which was qualified by the predicted food status by appetite interaction, *F*(1,48) = 9.11, *p* < 0.01, η**_p_^2^ = 0.16. No other effects were significant.

**FIGURE 1:**
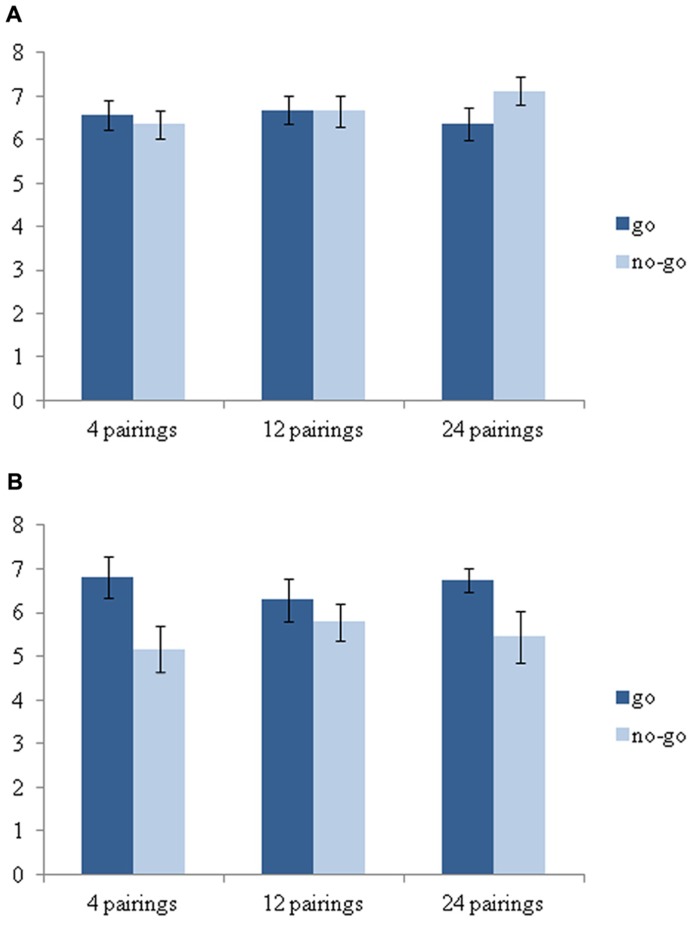
**(A)** Food evaluations as a function of amount of pairings and food status among participants with a relatively low appetite. **(B)** Food evaluations as a function of amount of pairings and food status among participants with a relatively high appetite. Error bars = SE.

Next, we examined the effect of food status (go vs. no-go) separately for participants in the low vs. high appetite condition. Within the low appetite condition there was no effect of food status, *F* < 1 (see **Figure 1A**). In contrast, the effect of food status in the high appetite condition was significant, *F*(1,48) = 11.02, *p* < 0.01, η _p_^2^ = 0.19. As can be seen in **Figure 1B**, the direction of the effect is in the same direction for each of the pairing conditions, indicating expected lower evaluations for no-go foods compared to go foods. Subsequent analyses showed that food associated with go cues (*M* = 6.61; SD = 1.04) was not significantly evaluated more positively than new food (*M* = 6.21; SD = 1.71), *F* < 1, and that food associated with no-go cues (*M* = 5.46; SD = 1.46) was not significantly evaluated more negatively than new food, *F*(1,48) = 1.69, ns. The absence of the latter effect may be due to the fact that the evaluations of new food involved only a single food stimulus rendering this test less powerful than the comparison with go foods. Additional analyses revealed that the effect of appetite on food evaluation was significant for no-go foods (*M*_no-go low appetite_ = 6.72; SD = 1.33 vs. *M*_no-go high appetite_ = 5.46; SD = 1.46), *F*(1,48) = 9.68, *p* < 0.01, η^2^ = 0.17, but not for go and new foods, *F*s < 1. This latter result suggest that participants in the high appetite condition devalued food that had been presented with no-go cues.

### FOOD CHOICE

Because we did not find an effect of amount of pairings on food evaluation, we collapsed the analyses of food choice over the factor amount of pairings. However, as can be seen in **Table 1**, the pattern of choice behavior within each pairing condition in the high appetite condition is in the predicted direction. Moreover, we did not analyze choices for the new foods because of two reasons. First, we obtained significant differences between go vs. no-go foods on evaluations, but not between go and no-go foods vs. new food. Second, the choice task contained three foods associated with go responses and three foods associated with no-go responses, but only one new food, leading to difficulties in comparing choices for new foods to the other types of foods within one analysis. The proportion of choices for new foods is presented in **Table 1**.

**Table 1:** Proportion of choices as a function of appetite, amount of pairings, and food status.

Amount of pairings Food status	4		12		24		0
	Go	No-go	Go	No-go	Go	No-go	New
Low appetite (*N* = 31)	0.42	0.23	0.45	0.39	0.35	0.61	0.48
High appetite (*N* = 19)	0.68	0.21	0.52	0.32	0.58	0.32	0.37

Food choice scores for the go foods and no-go foods could be 0, 1, 2, or 3. A repeated measures analysis with food status (go vs. no-go) as within-subject factor and appetite as between-subject factor on food choice, revealed the predicted interaction effect, *F*(1,48) = 4.02, *p* = 0.02, η**_p_^2^ = 0.10. Participants in the low appetite condition chose as much foods associated with no-go responses (*M* = 1.23; SD = 0.76) as foods associated with go responses (*M* = 1.23; SD = 0.72), *F* < 1, whereas participants in the high appetite condition chose less foods associated with no-go responses (*M* = 0.84; SD = 0.69) than with go responses (*M* = 1.79; SD = 0.79), *F*(1,48) = 8.81, *p* < 0.01, η _p_^2^ = 0.16. Additional analyses revealed that the effect of appetite was significant for choices for foods associated with go responses, *F*(1,48) = 6.76, *p* = 0.01, η _p_^2^ = 0.12, and marginally significant for foods associated with no-go responses, *F*(1,48) = 3.21, *p* = 0.08, η _p_^2^ = 0.06.

### MEDIATION ANALYSES

**FIGURE 2:**
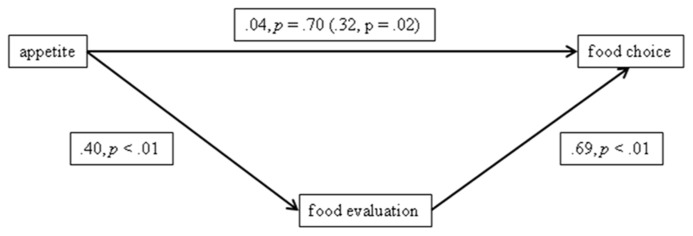
**Results of the mediation analyses according to the bootstrapping method proposed by [Bibr B16]**. Note: path values reflect standardized regression coefficients. The value in parentheses represents the total effect of appetite on food choice of the bootstrapping analyses. Values outside parentheses reflect the direct effects of the bootstrapping analyses.

Next, we tested whether the effects of appetite on choices for go vs. no-go food were mediated by food evaluation. First, we standardized the variables, and then we computed a difference score of food evaluation (go food evaluation – no-go food evaluation) and food choice (choices for go food – choices for no-go food). Next, we conducted a mediation analysis according to the bootstrapping method of Preacher and Hayes (2008) for estimating direct and indirect effects (with 1000 bootstrap resamples). As can be seen in **Figure 2** appetite is related to food evaluation and food evaluation is related to food choice. Importantly, the 95% confidence interval (CI) of the bootstrapping analysis for the indirect effect did not include zero (range: 0.1450–0.4789) suggesting that the direct influence of appetite on food choice is mediated by food evaluation (see **Figure 2**).

Importantly, in order to assess whether the mediation could be attributed to decreased evaluation of no-go food and/or increased evaluation of go food, we conducted two additional mediation analyses with evaluations of go and no-go foods as respective possible mediators instead of a difference score of food evaluation. These analyses revealed that evaluations of no-go food had a direct effect on food choice (standardized regression coefficient = -0.62, *p* < 0.01), and evaluations of no-go food mediated the direct effect of appetite on food choice in a similar fashion as the difference score (CI range for the indirect effect of evaluations of no-go food: 0.1021–0.4731). In contrast, evaluation of go foods did not have a direct effect on food choice (standardized regression coefficient = 0.15, *p* = 0.29) and did not mediate the effect (i.e., CI range for the indirect effect of go food evaluations, -0.301 to 0.1051, did include zero). These results indicate that decreased evaluations of no-go foods rather than any enhanced evaluations of go foods mediated food choice.

## DISCUSSION

The present study revealed that food evaluations among people with a relatively high appetite can be reduced by performing a go/no-go task that consistently presents these foods with no-go cues. Moreover, the reduced evaluations mediated subsequent food choice. As predicted, and consistent with previous work (Veling et al., 2013) this effect only occurred among participants with a relatively high appetite. This finding is consistent with the theory outlined in the introduction that stop signals lower evaluations of food only when people are sensitive to the impulse-evoking quality of such food. Thus, the present work provides important new insight into the mechanism by which choices for specific palatable foods can be reduced via stop signals: stop signals are effective to change food choice behavior via changes in food evaluation when people have an appetite. It should be noted, however, that based on the present results this conclusion is limited to foods that are used in the go/no-go task, and cannot be extended to (new) foods that were not present in the task.

Furthermore, results suggest that the influence of stop signals on food evaluations among participants with a high appetite is independent of the amount of pairings with the stop signals. However, the current study examined amount of pairings in an exploratory manner, and future work is needed to arrive at definite conclusions on this issue. Nonetheless, the fact that the decreased evaluations do not depend on extensive training is consistent with prior theorizing that the decrease in evaluations functions to release the impulse toward to food (Veling et al., 2008), which may occur already after one trial (e.g., Ferrey et al., 2012). That is, once a stop signal is presented in close temporal proximity of an impulse-eliciting stimulus the brain may immediately devalue the impulse-eliciting stimulus so that other stimuli can guide subsequent behavior.

The finding that amount of pairings does not affect the strength of the devaluation effect could also important from an applied perspective, because it suggests that there may be no need for extensive training per food stimulus to observe inhibitory effects of the stop signals on subsequent behavior. Nonetheless, it would be interesting to test in future work whether more extensive training leads to more lasting associations, and that the effect of amount of pairings could be observed with measurements of food evaluations and choice over longer time intervals.

Results showed that evaluations of the no-go foods did not significantly differ from evaluations of the new foods, opening up the possibility that the difference between no-go and go food evaluations is caused by increased evaluations of go foods instead of decreased evaluation of no-go foods. This explanation is unlikely, however, because previous work has shown that no-go stimuli are devalued and that evaluations of go stimuli do not increase (Fenske and Raymond, 2006; Veling et al., 2008). More important, evaluations of the no-go foods mediated the effect of appetite on food choice whereas evaluations of the go foods did not. This result suggests the change of food evaluation in the high appetite condition was caused by a change in no-go food evaluation, but not due to a change in go food evaluation. We think the fact that we did not obtain a difference between new and no-go foods may be caused by the fact that the evaluation of new foods was based on the evaluation of one food stimulus, which leads to lower reliability compared to the ratings of the no-go and go foods.

Related to this issue, results revealed that the effect of appetite was significant for choices of foods associated with go responses, but only marginally significant for choices associated with no-go responses. What do these data mean? We think the interpretation of these data is difficult. First, the food choices are not independent, so changes in one food category (e.g., no-go foods) may alter the selection of items in the other category (go foods). Furthermore, we have no theoretical basis for predicting whether reduced choices for no-go foods will result in more choices for go foods or in more choices for new foods. Accordingly, we still think the most parsimonious explanation for the complete pattern of results is that no-go foods were devalued based on the mediation analyses showing that evaluations of no-go foods mediated choice behavior whereas evaluations of go foods did not.

In the current study, as well as in our previous work (Veling et al., 2013), self-reported appetite as measured on a nine-point scale ranging from not at all to very high does not moderate the effect of stop signals, but is related to our appetite manipulation. Thus, it is clear across different studies that the current operationalization of appetite (i.e., before vs. after lunch) moderates effects of stop signals, whereas self-reported appetite does not. Why is this case? One reason we think may account for this finding is that people may be better in reporting straightforward overt behavior such as whether they had lunch already compared to their internal states (i.e., feelings of appetite). Future work may focus on the question how appetite can best be assessed with self-report measurements.

The present study has three additional limitations that are important to mention. First, because we did not provide participants with consequences of their choice, it could be that participants made hypothetical food choices. Second, we did not randomly assign participants to the different appetite conditions. Finally, we did not assess whether high appetite participants reacted more impulsively toward the foods than low appetite participants. Although this latter finding has been established with a manipulation of appetite that is identical to our manipulation (Seibt et al., 2007), it would still be important for future research to examine this issue within one experimental design. Note that the go/no-go task we used as a manipulation is not suitable to measure impulses, as participants make hardly any errors on this task, and because go responses upon presentation of food pictures are not good indicators of impulse strength (Veling and Aarts, 2011b).

One noteworthy aspect of the present work is that we used a within-subjects design to assess differences in evaluation and choice behavior of go and no-go foods, whereas previous work in the domain of eating and drinking behavior always used between-subject designs (e.g., Houben, 2011; Houben and Jansen, 2011; Veling et al., 2011, 2013; Jones and Field, 2013). Hence, the present research reveals that the go/no-go task can be used to target specific stimuli, but does not necessarily affect similar stimuli (e.g., all palatable foods). This insight suggests that the go/no-go task may be used to facilitate diets that require inhibition of choices for specific foods, and its use is not constrained to reducing food intake *per se*. Future work is needed to test this specific application further.

The present work converges well with earlier work showing that decreased evaluation of beer mediated decreased beer consumption after performing the go/no-go task in which beer images were presented with no-go cues (Houben et al., 2012). One difference between this previous study and the current study (apart from different stimulus materials and evaluation measure) is that in this previous study the mediator was measured only after collecting the consumption measure. The fact that the effect of the go/no-go task on behavior is mediated through reduced stimuli evaluations across different stimuli, dependent measures, and procedures, adds credibility to the broad conclusion that stop signals are an effective tool to change behavior through changing stimulus evaluations.

## Conflict of Interest Statement

The authors declare that the research was conducted in the absence of any commercial or financial relationships that could be construed as a potential conflict of interest.

## AUTHOR CONTRIBUTIONS

Harm Veling designed the experiment, analyzed the data, and wrote the manuscript. Henk Aarts and Wolfgang Stroebe provided significant contributions to the manuscript.

## Footnotes

1 We use the term training instead of conditioning to keep our terminology consistent with previous work (e.g., Wiers etal., 2010).
